# Cleidocranial dysostosis: a case report with clinical illustration

**DOI:** 10.11604/pamj.2021.38.368.29204

**Published:** 2021-04-15

**Authors:** Vanesa Villamil, Ramón Ruiz Pruneda, María Fernández Ibieta, César Salcedo Cánovas

**Affiliations:** 1 Pediatric Surgery Service, Hospital HM Nens, Barcelona, Spain,; 2 Pediatric Surgery Service, Virgen de la Arrixaca University Clinic Hospital, Murcia, Spain,; 3 Pediatric Surgery Service, Virgen Macarena University Hospital, Sevilla, Spain,; 4 Traumatology and Orthopaedic Service, Virgen de la Arrixaca University Clinic Hospital, Murcia, Spain

**Keywords:** Cleidocranial dysostosis, cleidocranial dysplasia, supernumerary teeth, wormian bones, case report

## Abstract

Cleidocranial Dysostosis or Dysplasia (CCD) is an infrequent clinical condition, with an autosomal dominant hereditary mode of inheritance. Triad lesions: multiple supernumerary teeth, partial or complete absence of the clavicles and open sagittal sutures and fontanelles. Nine-year-old female patient comes to our service for outpatient consultation with the main complaint of upper limbs mobility restriction with shoulders hypermotility. The chest X-ray showed partial absence of the clavicles and a cone-shaped thorax. The diagnosis of CCD was performed. Treatment of these patients requires a multidisciplinary approach which includes orthopaedic and dental corrections. The premature diagnosis allows a proper orientation for the treatment, offering a better life quality for the patient.

## Introduction

Cleidocranial Dysostosis or Dysplasia (CCD) is an infrequent clinical condition, with an autosomal dominant hereditary mode of inheritance [1,2]. Nevertheless, many cases occur spontaneously, with approximately one out of three patients having unaffected parents [3]. An estimated prevalence rate for CCD is one per million [4]. Both sexes are affected to an approximately equal extent [1]. Many CCD patients have the following triad lesions: multiple supernumerary teeth, partial or complete absence of the clavicles and open sagittal sutures and fontanelles. This triad is considered to be pathognomonic for diagnosis of CCD [5]. Other characteristics about this condition are delayed closure of fontanelles, brachycephaly, delayed eruption of permanent teeth, supernumerary teeth and morphological alterations of the upper and lower jaw, among others [6]. The aim of this article is to report a case of CCD with radiological illustration and also discuss the spectrum of clinical and radiographic features in CCD.

## Patient and observation

**Figure 1:**
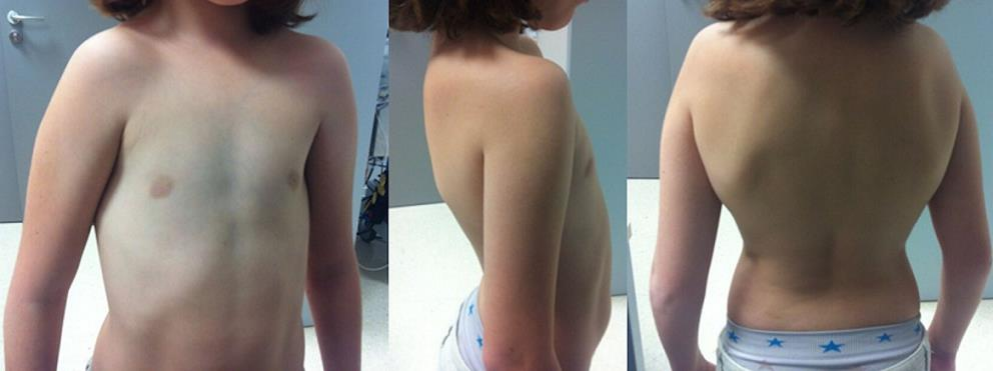
anterior view of the patient with cleidocranial dysostosis that shows the approximation of the shoulders

**Figure 2:**
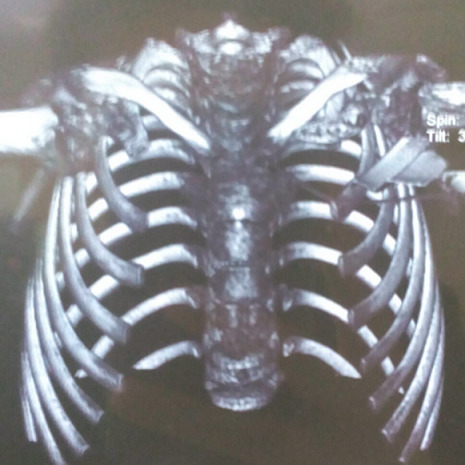
chest CT reconstruction showing the clavicular shortening

**Figure 3:**
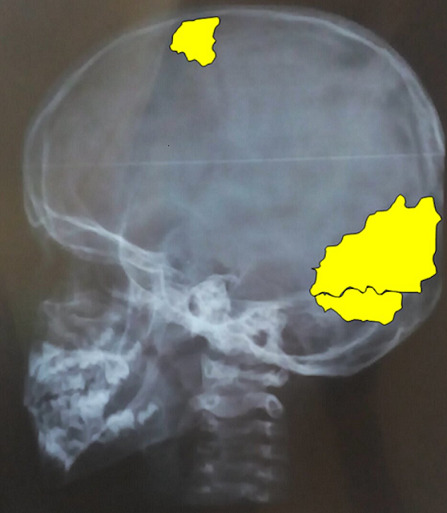
skull radiograph showing Wormian bones; note the frontal bossing and the small upper and lower jaw

**Figure 4:**
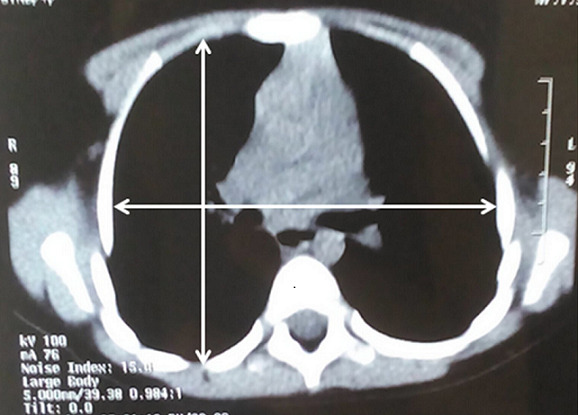
chest CT showing the overall decrease of transverse diameter of the rib cage

We present the case of a 9-year-old female patient that comes for outpatient consultations with the main complaint of upper limbs mobility restriction with shoulders hypermotility. During the anamnesis, the patient denied the existence of systemic or chronic diseases. Family history was uneventful. The facial appearance was hypertelorism and frontal bossing, with an increase in the cranial perimeter and a slightly wide neck. She was short in stature and had her humeral heads approximated anteriorly, with a dorsal hyperkyphosis and a lumbar hyperlordosis. She was unable to rise her elbow above her head and nor was achieved with passive movements, but, on the other hand, she was also able to approximate her shoulders in the midline (Figure 1). Her development and level of intelligence were normal and other systemic examination was unremarkable. The clinical features were suggestive of CCD. A chest computed tomography (CT) scan was performed and then a reconstruction was done showing clavicular shortening at the expense of aplasia of its acromial end (Figure 2) and a cone shaped thorax. Widened cranial sutures with Wormian bones were also seen in the skull radiography, in addition to a frontal and parietal bossing and a small upper and lower jaw (Figure 3). The CT scan also showed an overall decrease of the ribcage's transverse diameter (Figure 4), verticalization of the posterior costal arches, widening of the scapular's anterior margin and humeral heads displaced forward. Due to the clinical features and radiological findings, the diagnosis of CCD was performed. The patient was sent to Oral Surgery and Prosthodontic Department for a complete overhaul of the mouth and to traumatology and orthopaedic Department for orthopaedic treatment if it was indicated.

## Discussion

Cleidocranial Dysostosis (CCD) is rare in occurrence with an incidence of 1: 1.000.000 [7]. However, such low rate seems to be due to under diagnosis of relatively modest medical problems [4]. The phenotypic spectrum of CCD ranges from mildly affected individuals to severely affected patients. The most striking skeletal defects are hypoplasic or aplastic clavicles, late closure of the fontanelles, open skull sutures and multiple Wormian bones [8]. Here we present different clinical and radiological findings in these patients.

**Skull area:** cranial abnormalities include brachycephaly, a persistently open anterior fontanel, open skull sutures, prominent forehead, small sphenoid bones and calvarian thickening especially over the occiput and multiple Wormian bones [1] as seen in this case.

**Maxillary area:** the dental manifestations are very characteristic of CCD [1], despite our patient has a normal dentition, as in another case report publication [9]. A study of 14 patients with CCD, conclude that the second molar sign (eruption of the second molars despite persisting primary dentition) and spacing in the lower incisor area seems to appear consistently in patients with CCD [3]. Retention of the deciduous dentition with delayed eruption of permanent teeth is a relatively constant finding, predisposing to multiple tooth decays [1,8].

**Thoracic area:** the most frequent feature of this condition is the clavicle's anomaly, which could be either hypoplasic or aplastic clavicles [8]. Partial hypoplasia commonly involves the acromial end of the clavicles [10], as seen here, in our case. The thoracic cage is small, and bell or cone shaped with short, oblique ribs making the individual prone to chest infections [8].

**Pelvic area:** the pelvis is involved, showing characteristic changes, that is why this disease was postulate as “forme cleido cranio-pelvienne” by Crouzon and Buttier [10]. Pelvic features are delayed ossification with wide pubic symphysis, hypoplastic iliac wings, widened sacroiliac joints and a large femoral neck resulting in coxa vara [8,11].

**Complications:** the most common complications of CCD reported are pes planus, genu valgus, shoulder and hip dislocation, recurrent sinus infections, upper airway complications, recurrent ear infection, hearing loss, dental caries, osteomyelitis of the jaw bones and respiratory distress in early infancy which may be experienced because of narrow upper thoracic diameter [1,2,8,11]. However, even with these potential complications, the life expectancy in such patients is normal [11].

## Conclusion

Despite the variable expressivity, clinical and radiographic findings play a central role in the diagnosis of CCD. The signs and symptoms described here should serve as early markers to aid the paediatrician to suspect a disease in children with abnormal skeletal development, referring patients to specialized centres. The treatment of these patients requires a multidisciplinary approach which includes orthopaedic and dental corrections. A strict monitoring of bone mineral density must be done, in order to assess a preventive treatment for osteoporosis. The premature diagnosis allows a proper orientation for the treatment, offering a better life quality for the patient.
